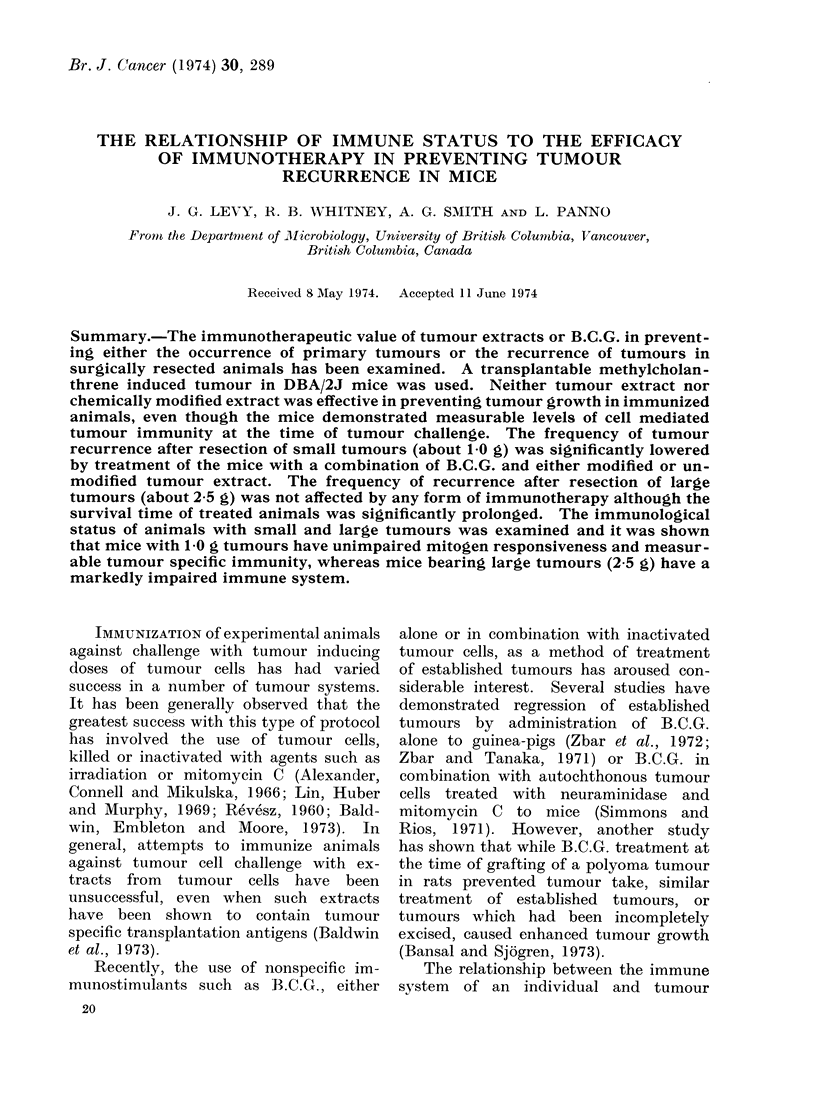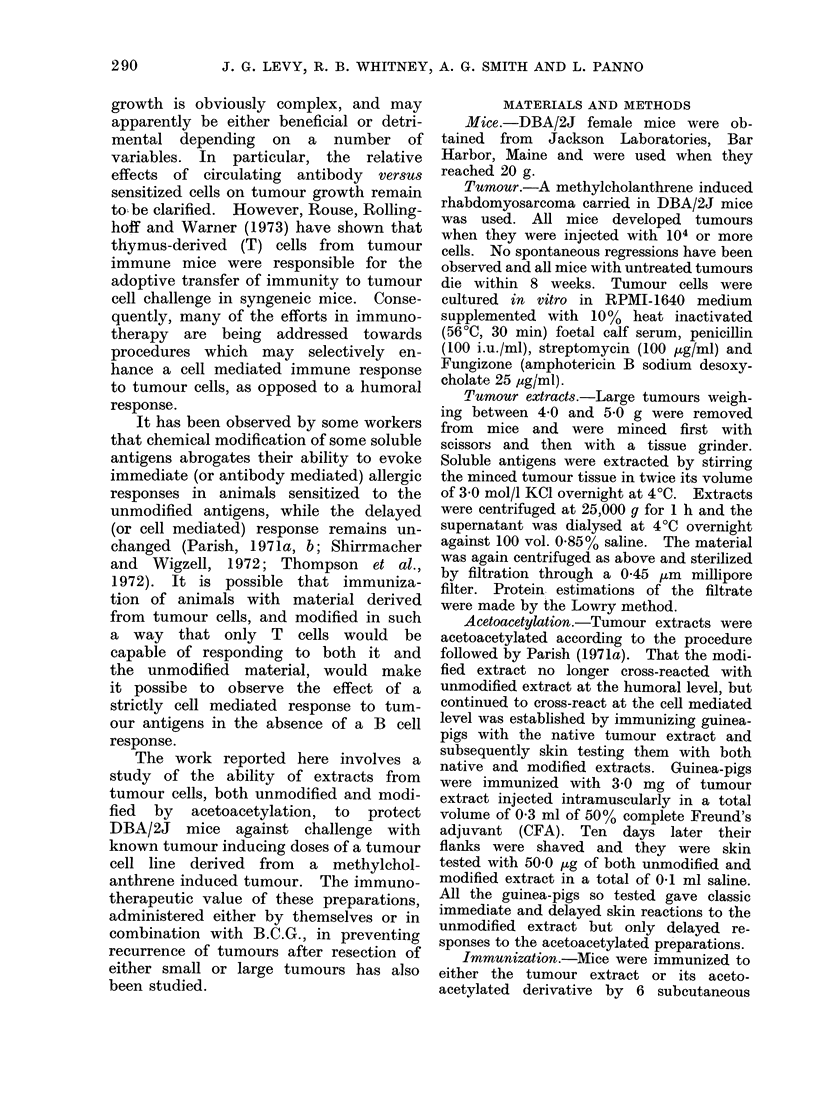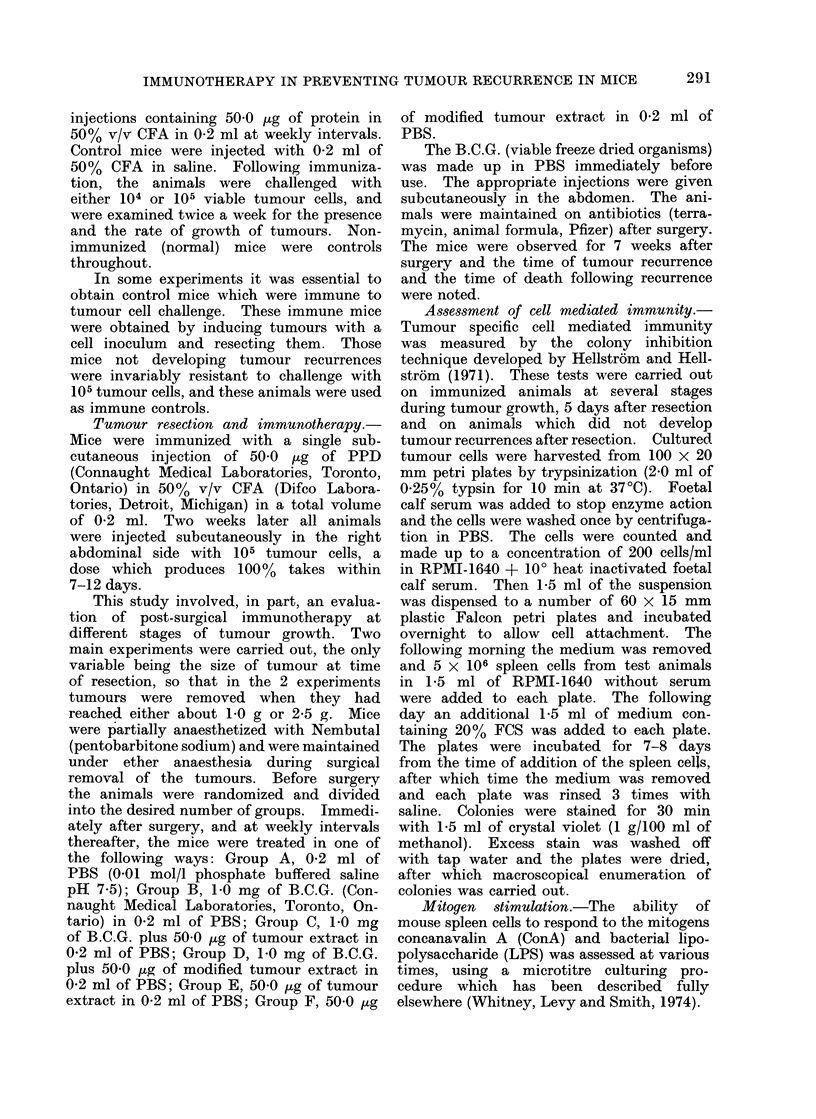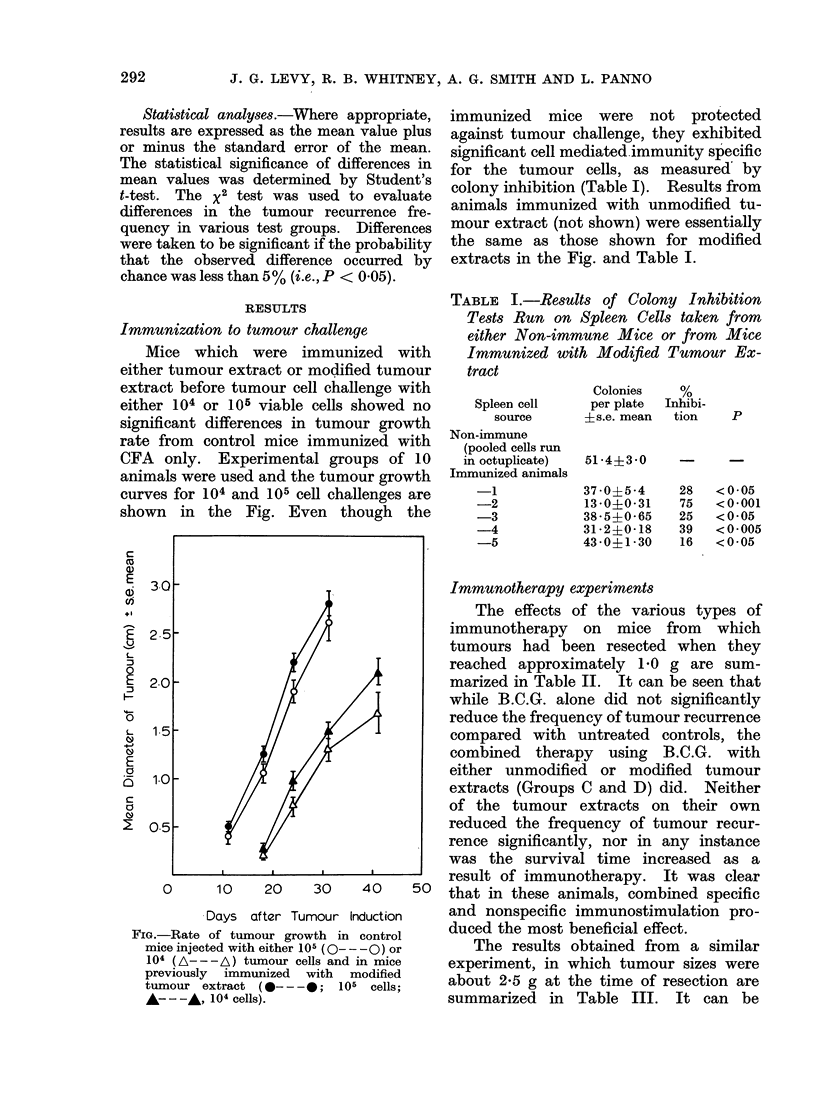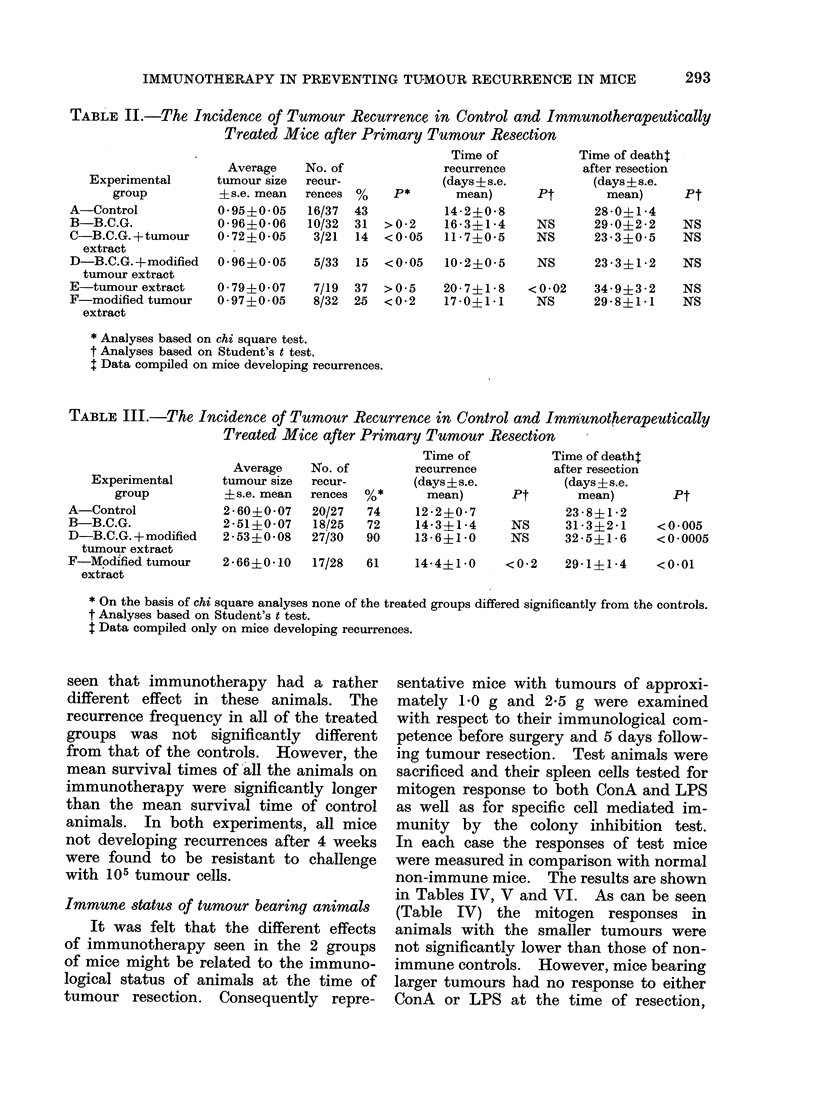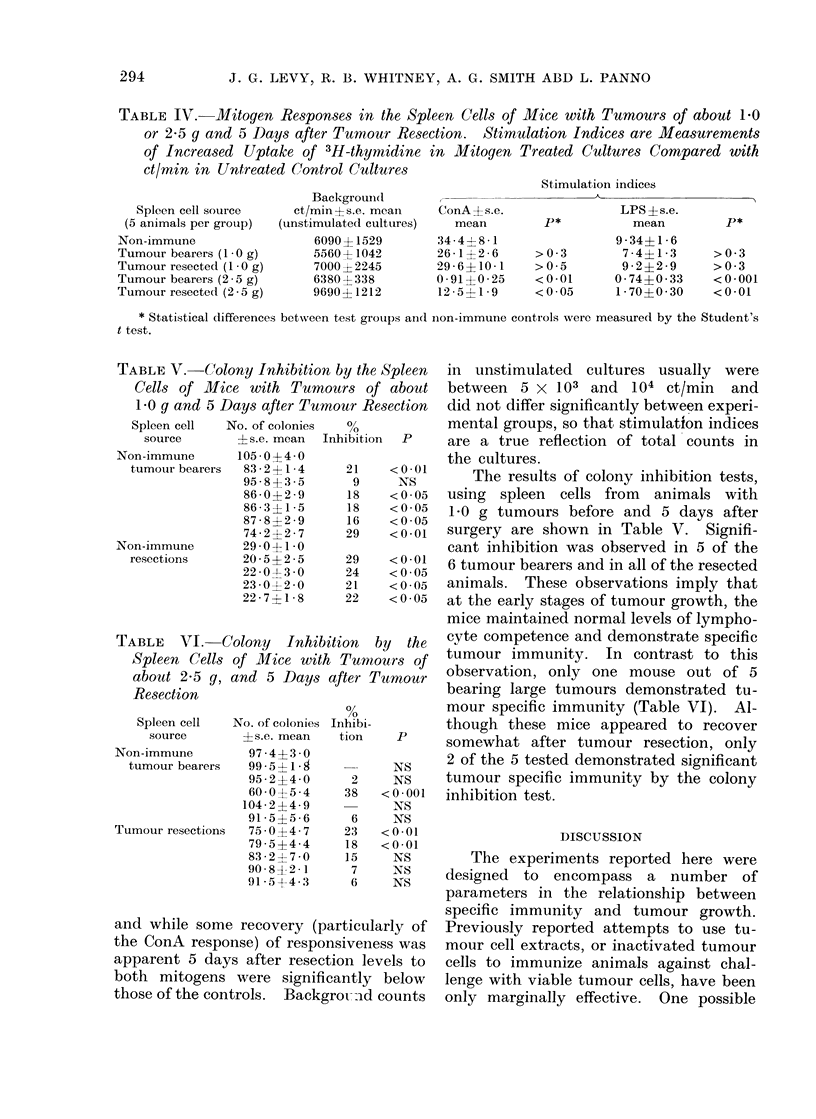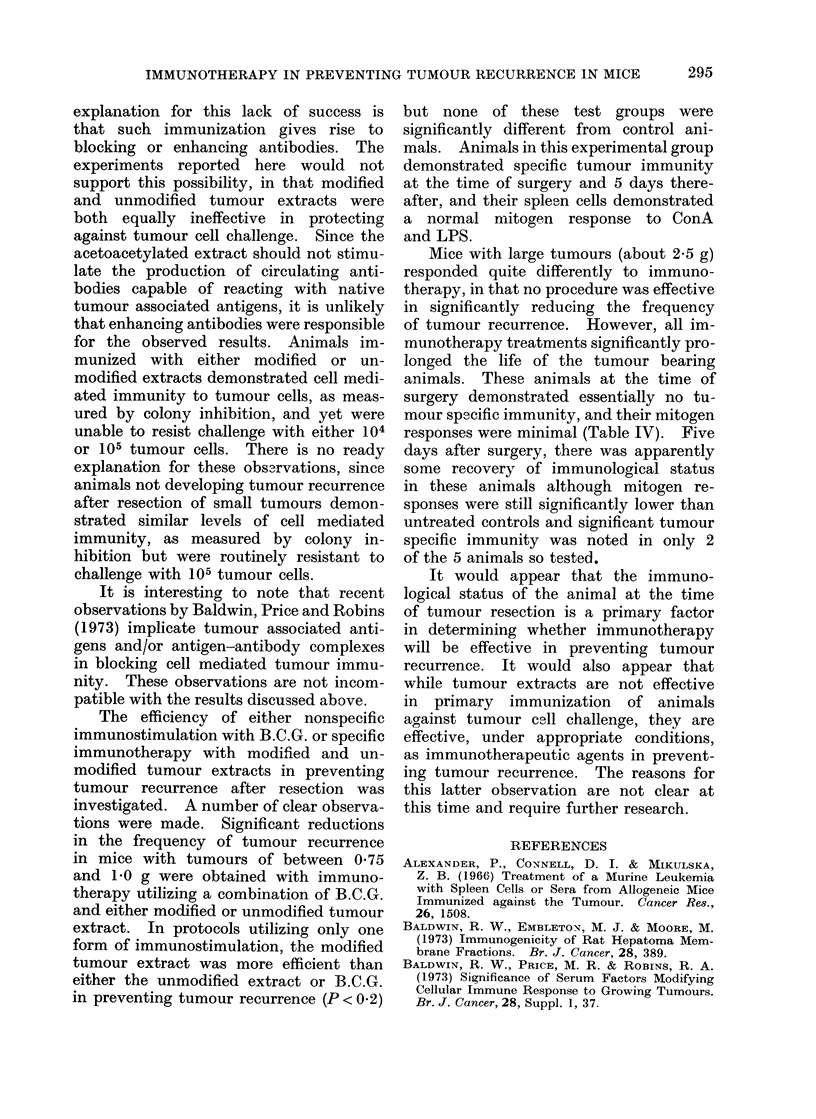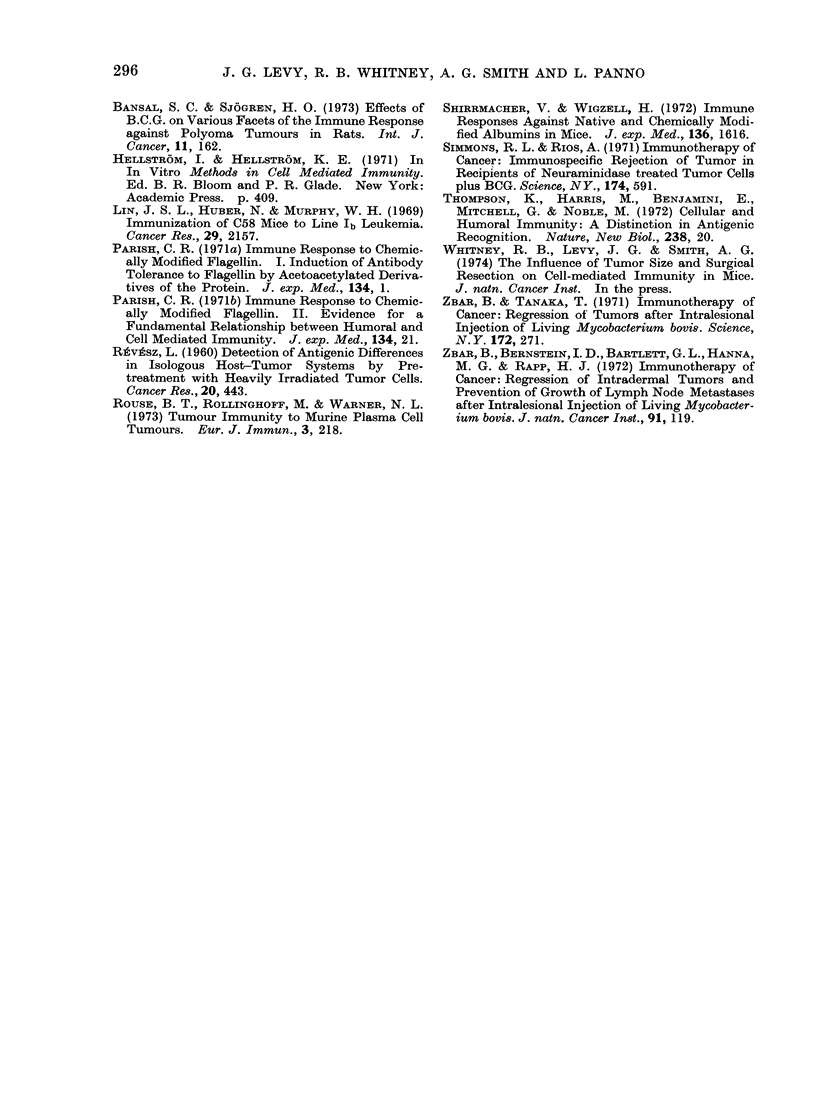# The Relationship of Immune Status to the Efficacy of Immunotherapy in Preventing Tumour Recurrence in Mice

**DOI:** 10.1038/bjc.1974.195

**Published:** 1974-10

**Authors:** J. G. Levy, R. B. Whitney, A. G. Smith, L. Panno

## Abstract

The immunotherapeutic value of tumour extracts or B.C.G. in preventing either the occurrence of primary tumours or the recurrence of tumours in surgically resected animals has been examined. A transplantable methylcholanthrene induced tumour in DBA/2J mice was used. Neither tumour extract nor chemically modified extract was effective in preventing tumour growth in immunized animals, even though the mice demonstrated measurable levels of cell mediated tumour immunity at the time of tumour challenge. The frequency of tumour recurrence after resection of small tumours (about 1·0 g) was significantly lowered by treatment of the mice with a combination of B.C.G. and either modified or unmodified tumour extract. The frequency of recurrence after resection of large tumours (about 2·5 g) was not affected by any form of immunotherapy although the survival time of treated animals was significantly prolonged. The immunological status of animals with small and large tumours was examined and it was shown that mice with 1·0 g tumours have unimpaired mitogen responsiveness and measurable tumour specific immunity, whereas mice bearing large tumours (2·5 g) have a markedly impaired immune system.


Br. J. Cancer (1974) **30**, 289

### THE RELATIONSHIP OF IMMUNE STATUS TO THE EFFICACY OF IMMUNOTHERAPY IN PREVENTING TUMOUR RECURRENCE IN MICE

J. G. LEVY, R. B. WHITNEY, A. G. SMITH AND L. PANNO

*From the Department of Microbiology, University of British Columbia, Vancouver, British Columbia, Canada*

Received 8 May 1974. Accepted 11 June 1974

**Summary.—The immunotherapeutic value of tumour extracts or B.C.G. in preventing either the occurrence of primary tumours or the recurrence of tumours in surgically resected animals has been examined. A transplantable methylcholanthrene induced tumour in DBA/2J mice was used. Neither tumour extract nor chemically modified extract was effective in preventing tumour growth in immunized animals, even though the mice demonstrated measurable levels of cell mediated tumour immunity at the time of tumour challenge. The frequency of tumour recurrence after resection of small tumours (about 1·0 g) was significantly lowered by treatment of the mice with a combination of B.C.G. and either modified or unmodified tumour extract. The frequency of recurrence after resection of large tumours (about 2·5 g) was not affected by any form of immunotherapy although the survival time of treated animals was significantly prolonged. The immunological status of animals with small and large tumours was examined and it was shown that mice with 1·0 g tumours have unimpaired mitogen responsiveness and measurable tumour specific immunity, whereas mice bearing large tumours (2·5 g) have a markedly impaired immune system.**

IMMUNIZATION of experimental animals against challenge with tumour inducing doses of tumour cells has had varied success in a number of tumour systems. It has been generally observed that the greatest success with this type of protocol has involved the use of tumour cells, killed or inactivated with agents such as irradiation or mitomycin C (Alexander, Connell and Mikulska, 1966; Lin, Huber and Murphy, 1969; Révész, 1960; Baldwin, Embleton and Moore, 1973). In general, attempts to immunize animals against tumour cell challenge with extracts from tumour cells have been unsuccessful, even when such extracts have been shown to contain tumour specific transplantation antigens (Baldwin *et al.*, 1973).

Recently, the use of nonspecific immunostimulants such as B.C.G., either alone or in combination with inactivated tumour cells, as a method of treatment of established tumours has aroused considerable interest. Several studies have demonstrated regression of established tumours by administration of B.C.G. alone to guinea-pigs (Zbar *et al.*, 1972; Zbar and Tanaka, 1971) or B.C.G. in combination with autochthonous tumour cells treated with neuraminidase and mitomycin C to mice (Simmons and Rios, 1971). However, another study has shown that while B.C.G. treatment at the time of grafting of a polyoma tumour in rats prevented tumour take, similar treatment of established tumours, or tumours which had been incompletely excised, caused enhanced tumour growth (Bansal and Sjögren, 1973).

The relationship between the immune system of an individual and tumour growth is obviously complex, and may apparently be either beneficial or detrimental depending on a number of variables. In particular, the relative effects of circulating antibody *versus* sensitized cells on tumour growth remain to be clarified. However, Rouse, Rollinghoff and Warner (1973) have shown that thymus-derived (T) cells from tumour immune mice were responsible for the adoptive transfer of immunity to tumour cell challenge in syngeneic mice. Consequently, many of the efforts in immunotherapy are being addressed towards procedures which may selectively enhance a cell mediated immune response to tumour cells, as opposed to a humoral response.

It has been observed by some workers that chemical modification of some soluble antigens abrogates their ability to evoke immediate (or antibody mediated) allergic responses in animals sensitized to the unmodified antigens, while the delayed (or cell mediated) response remains unchanged (Parish, 1971*a*, *b*; Shirrmacher and Wigzell, 1972; Thompson *et al.*, 1972). It is possible that immunization of animals with material derived from tumour cells, and modified in such a way that only T cells would be capable of responding to both it and the unmodified material, would make it possibe to observe the effect of a strictly cell mediated response to tumour antigens in the absence of a B cell response.

The work reported here involves a study of the ability of extracts from tumour cells, both unmodified and modified by acetoacetylation, to protect DBA/2J mice against challenge with known tumour inducing doses of a tumour cell line derived from a methylcholanthrene induced tumour. The immunotherapeutic value of these preparations, administered either by themselves or in combination with B.C.G., in preventing recurrence of tumours after resection of either small or large tumours has also been studied.

### MATERIALS AND METHODS

*Mice*.—DBA/2J female mice were obtained from Jackson Laboratories, Bar Harbor, Maine and were used when they reached 20 g.

*Tumour*.—A methylcholanthrene induced rhabdomyosarcoma carried in DBA/2J mice was used. All mice developed tumours when they were injected with $10^4$ or more cells. No spontaneous regressions have been observed and all mice with untreated tumours die within 8 weeks. Tumour cells were cultured *in vitro* in RPMI-1640 medium supplemented with 10% heat inactivated (56°C, 30 min) foetal calf serum, penicillin (100 i.u./ml), streptomycin (100 $\mu$g/ml) and Fungizone (amphotericin B sodium desoxycholate 25 $\mu$g/ml).

*Tumour extracts*.—Large tumours weighing between 4·0 and 5·0 g were removed from mice and were minced first with scissors and then with a tissue grinder. Soluble antigens were extracted by stirring the minced tumour tissue in twice its volume of 3·0 mol/l KCl overnight at 4°C. Extracts were centrifuged at 25,000 *g* for 1 h and the supernatant was dialysed at 4°C overnight against 100 vol. 0·85% saline. The material was again centrifuged as above and sterilized by filtration through a 0·45 $\mu$m millipore filter. Protein estimations of the filtrate were made by the Lowry method.

*Acetoacetylation*.—Tumour extracts were acetoacetylated according to the procedure followed by Parish (1971*a*). That the modified extract no longer cross-reacted with unmodified extract at the humoral level, but continued to cross-react at the cell mediated level was established by immunizing guinea-pigs with the native tumour extract and subsequently skin testing them with both native and modified extracts. Guinea-pigs were immunized with 3·0 mg of tumour extract injected intramuscularly in a total volume of 0·3 ml of 50% complete Freund's adjuvant (CFA). Ten days later their flanks were shaved and they were skin tested with 50·0 $\mu$g of both unmodified and modified extract in a total of 0·1 ml saline. All the guinea-pigs so tested gave classic immediate and delayed skin reactions to the unmodified extract but only delayed responses to the acetoacetylated preparations.

*Immunization*.—Mice were immunized to either the tumour extract or its acetoacetylated derivative by 6 subcutaneous injections containing 50·0 μg of protein in 50% v/v CFA in 0·2 ml at weekly intervals. Control mice were injected with 0·2 ml of 50% CFA in saline. Following immunization, the animals were challenged with either $10^4$ or $10^5$ viable tumour cells, and were examined twice a week for the presence and the rate of growth of tumours. Non-immunized (normal) mice were controls throughout.

In some experiments it was essential to obtain control mice which were immune to tumour cell challenge. These immune mice were obtained by inducing tumours with a cell inoculum and resecting them. Those mice not developing tumour recurrences were invariably resistant to challenge with $10^5$ tumour cells, and these animals were used as immune controls.

*Tumour resection and immunotherapy.*—Mice were immunized with a single subcutaneous injection of 50·0 μg of PPD (Connaught Medical Laboratories, Toronto, Ontario) in 50% v/v CFA (Difco Laboratories, Detroit, Michigan) in a total volume of 0·2 ml. Two weeks later all animals were injected subcutaneously in the right abdominal side with $10^5$ tumour cells, a dose which produces 100% takes within 7–12 days.

This study involved, in part, an evaluation of post-surgical immunotherapy at different stages of tumour growth. Two main experiments were carried out, the only variable being the size of tumour at time of resection, so that in the 2 experiments tumours were removed when they had reached either about 1·0 g or 2·5 g. Mice were partially anaesthetized with Nembutal (pentobarbitone sodium) and were maintained under ether anaesthesia during surgical removal of the tumours. Before surgery the animals were randomized and divided into the desired number of groups. Immediately after surgery, and at weekly intervals thereafter, the mice were treated in one of the following ways: Group A, 0·2 ml of PBS (0·01 mol/l phosphate buffered saline pH 7·5); Group B, 1·0 mg of B.C.G. (Connaught Medical Laboratories, Toronto, Ontario) in 0·2 ml of PBS; Group C, 1·0 mg of B.C.G. plus 50·0 μg of tumour extract in 0·2 ml of PBS; Group D, 1·0 mg of B.C.G. plus 50·0 μg of modified tumour extract in 0·2 ml of PBS; Group E, 50·0 μg of tumour extract in 0·2 ml of PBS; Group F, 50·0 μg of modified tumour extract in 0·2 ml of PBS.

The B.C.G. (viable freeze dried organisms) was made up in PBS immediately before use. The appropriate injections were given subcutaneously in the abdomen. The animals were maintained on antibiotics (terramycin, animal formula, Pfizer) after surgery. The mice were observed for 7 weeks after surgery and the time of tumour recurrence and the time of death following recurrence were noted.

*Assessment of cell mediated immunity.*—Tumour specific cell mediated immunity was measured by the colony inhibition technique developed by Hellström and Hellström (1971). These tests were carried out on immunized animals at several stages during tumour growth, 5 days after resection and on animals which did not develop tumour recurrences after resection. Cultured tumour cells were harvested from 100 × 20 mm petri plates by trypsinization (2·0 ml of 0·25% typsin for 10 min at 37°C). Foetal calf serum was added to stop enzyme action and the cells were washed once by centrifugation in PBS. The cells were counted and made up to a concentration of 200 cells/ml in RPMI-1640 + 10° heat inactivated foetal calf serum. Then 1·5 ml of the suspension was dispensed to a number of 60 × 15 mm plastic Falcon petri plates and incubated overnight to allow cell attachment. The following morning the medium was removed and $5 \times 10^6$ spleen cells from test animals in 1·5 ml of RPMI-1640 without serum were added to each plate. The following day an additional 1·5 ml of medium containing 20% FCS was added to each plate. The plates were incubated for 7–8 days from the time of addition of the spleen cells, after which time the medium was removed and each plate was rinsed 3 times with saline. Colonies were stained for 30 min with 1·5 ml of crystal violet (1 g/100 ml of methanol). Excess stain was washed off with tap water and the plates were dried, after which macroscopical enumeration of colonies was carried out.

*Mitogen stimulation.*—The ability of mouse spleen cells to respond to the mitogens concanavalin A (ConA) and bacterial lipopolysaccharide (LPS) was assessed at various times, using a microtitre culturing procedure which has been described fully elsewhere (Whitney, Levy and Smith, 1974).

*Statistical analyses.*—Where appropriate, results are expressed as the mean value plus or minus the standard error of the mean. The statistical significance of differences in mean values was determined by Student's *t*-test. The $\chi^2$ test was used to evaluate differences in the tumour recurrence frequency in various test groups. Differences were taken to be significant if the probability that the observed difference occurred by chance was less than 5% (*i.e.*, $P < 0{\cdot}05$).

### RESULTS

### *Immunization to tumour challenge*

Mice which were immunized with either tumour extract or modified tumour extract before tumour cell challenge with either $10^4$ or $10^5$ viable cells showed no significant differences in tumour growth rate from control mice immunized with CFA only. Experimental groups of 10 animals were used and the tumour growth curves for $10^4$ and $10^5$ cell challenges are shown in the Fig. Even though the

FIG.—Rate of tumour growth in control mice injected with either $10^5$ (○– – –○) or $10^4$ (△– – –△) tumour cells and in mice previously immunized with modified tumour extract (●– – –●; $10^5$ cells; ▲– – –▲, $10^4$ cells).

immunized mice were not protected against tumour challenge, they exhibited significant cell mediated immunity specific for the tumour cells, as measured by colony inhibition (Table I). Results from animals immunized with unmodified tumour extract (not shown) were essentially the same as those shown for modified extracts in the Fig. and Table I.

TABLE I.—*Results of Colony Inhibition Tests Run on Spleen Cells taken from either Non-immune Mice or from Mice Immunized with Modified Tumour Extract*

| Spleen cell source | Colonies per plate ±s.e. mean | % Inhibition | *P* |
|---|---|---|---|
| Non-immune (pooled cells run in octuplicate) | 51·4±3·0 | — | — |
| Immunized animals | | | |
| —1 | 37·0±5·4 | 28 | <0·05 |
| —2 | 13·0±0·31 | 75 | <0·001 |
| —3 | 38·5±0·65 | 25 | <0·05 |
| —4 | 31·2±0·18 | 39 | <0·005 |
| —5 | 43·0±1·30 | 16 | <0·05 |

### *Immunotherapy experiments*

The effects of the various types of immunotherapy on mice from which tumours had been resected when they reached approximately 1·0 g are summarized in Table II. It can be seen that while B.C.G. alone did not significantly reduce the frequency of tumour recurrence compared with untreated controls, the combined therapy using B.C.G. with either unmodified or modified tumour extracts (Groups C and D) did. Neither of the tumour extracts on their own reduced the frequency of tumour recurrence significantly, nor in any instance was the survival time increased as a result of immunotherapy. It was clear that in these animals, combined specific and nonspecific immunostimulation produced the most beneficial effect.

The results obtained from a similar experiment, in which tumour sizes were about 2·5 g at the time of resection are summarized in Table III. It can be

TABLE II.—*The Incidence of Tumour Recurrence in Control and Immunotherapeutically Treated Mice after Primary Tumour Resection*

| Experimental group | Average tumour size ±s.e. mean | No. of recurrences | % | P* | Time of recurrence (days±s.e. mean) | P† | Time of death‡ after resection (days±s.e. mean) | P† |
|---|---|---|---|---|---|---|---|---|
| A—Control | 0·95±0·05 | 16/37 | 43 | | 14·2±0·8 | | 28·0±1·4 | |
| B—B.C.G. | 0·96±0·06 | 10/32 | 31 | >0·2 | 16·3±1·4 | NS | 29·0±2·2 | NS |
| C—B.C.G.+tumour extract | 0·72±0·05 | 3/21 | 14 | <0·05 | 11·7±0·5 | NS | 23·3±0·5 | NS |
| D—B.C.G.+modified tumour extract | 0·96±0·05 | 5/33 | 15 | <0·05 | 10·2±0·5 | NS | 23·3±1·2 | NS |
| E—tumour extract | 0·79±0·07 | 7/19 | 37 | >0·5 | 20·7±1·8 | <0·02 | 34·9±3·2 | NS |
| F—modified tumour extract | 0·97±0·05 | 8/32 | 25 | <0·2 | 17·0±1·1 | NS | 29·8±1·1 | NS |

* Analyses based on *chi* square test.
† Analyses based on Student's *t* test.
‡ Data compiled on mice developing recurrences.

TABLE III.—*The Incidence of Tumour Recurrence in Control and Immunotherapeutically Treated Mice after Primary Tumour Resection*

| Experimental group | Average tumour size ±s.e. mean | No. of recurrences | %* | Time of recurrence (days±s.e. mean) | P† | Time of death‡ after resection (days±s.e. mean) | P† |
|---|---|---|---|---|---|---|---|
| A—Control | 2·60±0·07 | 20/27 | 74 | 12·2±0·7 | | 23·8±1·2 | |
| B—B.C.G. | 2·51±0·07 | 18/25 | 72 | 14·3±1·4 | NS | 31·3±2·1 | <0·005 |
| D—B.C.G.+modified tumour extract | 2·53±0·08 | 27/30 | 90 | 13·6±1·0 | NS | 32·5±1·6 | <0·0005 |
| F—Modified tumour extract | 2·66±0·10 | 17/28 | 61 | 14·4±1·0 | <0·2 | 29·1±1·4 | <0·01 |

* On the basis of *chi* square analyses none of the treated groups differed significantly from the controls.
† Analyses based on Student's *t* test.
‡ Data compiled only on mice developing recurrences.

seen that immunotherapy had a rather different effect in these animals. The recurrence frequency in all of the treated groups was not significantly different from that of the controls. However, the mean survival times of all the animals on immunotherapy were significantly longer than the mean survival time of control animals. In both experiments, all mice not developing recurrences after 4 weeks were found to be resistant to challenge with $10^5$ tumour cells.

*Immune status of tumour bearing animals*

It was felt that the different effects of immunotherapy seen in the 2 groups of mice might be related to the immunological status of animals at the time of tumour resection. Consequently representative mice with tumours of approximately 1·0 g and 2·5 g were examined with respect to their immunological competence before surgery and 5 days following tumour resection. Test animals were sacrificed and their spleen cells tested for mitogen response to both ConA and LPS as well as for specific cell mediated immunity by the colony inhibition test. In each case the responses of test mice were measured in comparison with normal non-immune mice. The results are shown in Tables IV, V and VI. As can be seen (Table IV) the mitogen responses in animals with the smaller tumours were not significantly lower than those of non-immune controls. However, mice bearing larger tumours had no response to either ConA or LPS at the time of resection,

TABLE IV.—*Mitogen Responses in the Spleen Cells of Mice with Tumours of about* 1·0 *or* 2·5 *g and* 5 *Days after Tumour Resection. Stimulation Indices are Measurements of Increased Uptake of* $^3$*H-thymidine in Mitogen Treated Cultures Compared with ct/min in Untreated Control Cultures*

| Spleen cell source (5 animals per group) | Background ct/min ± s.e. mean (unstimulated cultures) | Stimulation indices: ConA ± s.e. mean | P* | LPS ± s.e. mean | P* |
|---|---|---|---|---|---|
| Non-immune | 6090 ± 1529 | 34·4 ± 8·1 | | 9·34 ± 1·6 | |
| Tumour bearers (1·0 g) | 5560 ± 1042 | 26·1 ± 2·6 | > 0·3 | 7·4 ± 1·3 | > 0·3 |
| Tumour resected (1·0 g) | 7000 ± 2245 | 29·6 ± 10·1 | > 0·5 | 9·2 ± 2·9 | > 0·3 |
| Tumour bearers (2·5 g) | 6380 ± 338 | 0·91 ± 0·25 | < 0·01 | 0·74 ± 0·33 | < 0·001 |
| Tumour resected (2·5 g) | 9690 ± 1212 | 12·5 ± 1·9 | < 0·05 | 1·70 ± 0·30 | < 0·01 |

* Statistical differences between test groups and non-immune controls were measured by the Student's *t* test.

TABLE V.—*Colony Inhibition by the Spleen Cells of Mice with Tumours of about* 1·0 *g and* 5 *Days after Tumour Resection*

| Spleen cell source | No. of colonies ± s.e. mean | % Inhibition | P |
|---|---|---|---|
| Non-immune | 105·0 ± 4·0 | | |
| tumour bearers | 83·2 ± 1·4 | 21 | < 0·01 |
| | 95·8 ± 3·5 | 9 | NS |
| | 86·0 ± 2·9 | 18 | < 0·05 |
| | 86·3 ± 1·5 | 18 | < 0·05 |
| | 87·8 ± 2·9 | 16 | < 0·05 |
| | 74·2 ± 2·7 | 29 | < 0·01 |
| Non-immune | 29·0 ± 1·0 | | |
| resections | 20·5 ± 2·5 | 29 | < 0·01 |
| | 22·0 ± 3·0 | 24 | < 0·05 |
| | 23·0 ± 2·0 | 21 | < 0·05 |
| | 22·7 ± 1·8 | 22 | < 0·05 |

TABLE VI.—*Colony Inhibition by the Spleen Cells of Mice with Tumours of about* 2·5 *g, and* 5 *Days after Tumour Resection*

| Spleen cell source | No. of colonies ± s.e. mean | % Inhibition | P |
|---|---|---|---|
| Non-immune | 97·4 ± 3·0 | | |
| tumour bearers | 99·5 ± 1·8 | — | NS |
| | 95·2 ± 4·0 | 2 | NS |
| | 60·0 ± 5·4 | 38 | < 0·001 |
| | 104·2 ± 4·9 | — | NS |
| | 91·5 ± 5·6 | 6 | NS |
| Tumour resections | 75·0 ± 4·7 | 23 | < 0·01 |
| | 79·5 ± 4·4 | 18 | < 0·01 |
| | 83·2 ± 7·0 | 15 | NS |
| | 90·8 ± 2·1 | 7 | NS |
| | 91·5 ± 4·3 | 6 | NS |

and while some recovery (particularly of the ConA response) of responsiveness was apparent 5 days after resection levels to both mitogens were significantly below those of the controls. Background counts in unstimulated cultures usually were between $5 \times 10^3$ and $10^4$ ct/min and did not differ significantly between experimental groups, so that stimulation indices are a true reflection of total counts in the cultures.

The results of colony inhibition tests, using spleen cells from animals with 1·0 g tumours before and 5 days after surgery are shown in Table V. Significant inhibition was observed in 5 of the 6 tumour bearers and in all of the resected animals. These observations imply that at the early stages of tumour growth, the mice maintained normal levels of lymphocyte competence and demonstrate specific tumour immunity. In contrast to this observation, only one mouse out of 5 bearing large tumours demonstrated tumour specific immunity (Table VI). Although these mice appeared to recover somewhat after tumour resection, only 2 of the 5 tested demonstrated significant tumour specific immunity by the colony inhibition test.

### DISCUSSION

The experiments reported here were designed to encompass a number of parameters in the relationship between specific immunity and tumour growth. Previously reported attempts to use tumour cell extracts, or inactivated tumour cells to immunize animals against challenge with viable tumour cells, have been only marginally effective. One possible explanation for this lack of success is that such immunization gives rise to blocking or enhancing antibodies. The experiments reported here would not support this possibility, in that modified and unmodified tumour extracts were both equally ineffective in protecting against tumour cell challenge. Since the acetoacetylated extract should not stimulate the production of circulating antibodies capable of reacting with native tumour associated antigens, it is unlikely that enhancing antibodies were responsible for the observed results. Animals immunized with either modified or unmodified extracts demonstrated cell mediated immunity to tumour cells, as measured by colony inhibition, and yet were unable to resist challenge with either $10^4$ or $10^5$ tumour cells. There is no ready explanation for these observations, since animals not developing tumour recurrence after resection of small tumours demonstrated similar levels of cell mediated immunity, as measured by colony inhibition but were routinely resistant to challenge with $10^5$ tumour cells.

It is interesting to note that recent observations by Baldwin, Price and Robins (1973) implicate tumour associated antigens and/or antigen–antibody complexes in blocking cell mediated tumour immunity. These observations are not incompatible with the results discussed above.

The efficiency of either nonspecific immunostimulation with B.C.G. or specific immunotherapy with modified and unmodified tumour extracts in preventing tumour recurrence after resection was investigated. A number of clear observations were made. Significant reductions in the frequency of tumour recurrence in mice with tumours of between 0·75 and 1·0 g were obtained with immunotherapy utilizing a combination of B.C.G. and either modified or unmodified tumour extract. In protocols utilizing only one form of immunostimulation, the modified tumour extract was more efficient than either the unmodified extract or B.C.G. in preventing tumour recurrence ($P < 0{\cdot}2$) but none of these test groups were significantly different from control animals. Animals in this experimental group demonstrated specific tumour immunity at the time of surgery and 5 days thereafter, and their spleen cells demonstrated a normal mitogen response to ConA and LPS.

Mice with large tumours (about 2·5 g) responded quite differently to immunotherapy, in that no procedure was effective in significantly reducing the frequency of tumour recurrence. However, all immunotherapy treatments significantly prolonged the life of the tumour bearing animals. These animals at the time of surgery demonstrated essentially no tumour specific immunity, and their mitogen responses were minimal (Table IV). Five days after surgery, there was apparently some recovery of immunological status in these animals although mitogen responses were still significantly lower than untreated controls and significant tumour specific immunity was noted in only 2 of the 5 animals so tested.

It would appear that the immunological status of the animal at the time of tumour resection is a primary factor in determining whether immunotherapy will be effective in preventing tumour recurrence. It would also appear that while tumour extracts are not effective in primary immunization of animals against tumour cell challenge, they are effective, under appropriate conditions, as immunotherapeutic agents in preventing tumour recurrence. The reasons for this latter observation are not clear at this time and require further research.

### REFERENCES

Alexander, P., Connell, D. I. & Mikulska, Z. B. (1966) Treatment of a Murine Leukemia with Spleen Cells or Sera from Allogeneic Mice Immunized against the Tumour. *Cancer Res.*, **26**, 1508.

Baldwin, R. W., Embleton, M. J. & Moore, M. (1973) Immunogenicity of Rat Hepatoma Membrane Fractions. *Br. J. Cancer*, **28**, 389.

Baldwin, R. W., Price, M. R. & Robins, R. A. (1973) Significance of Serum Factors Modifying Cellular Immune Response to Growing Tumours. *Br. J. Cancer*, **28**, Suppl. 1, 37.

BANSAL, S. C. & SJÖGREN, H. O. (1973) Effects of B.C.G. on Various Facets of the Immune Response against Polyoma Tumours in Rats. *Int. J. Cancer*, **11**, 162.

HELLSTRÖM, I. & HELLSTRÖM, K. E. (1971) In In Vitro *Methods in Cell Mediated Immunity*. Ed. B. R. Bloom and P. R. Glade. New York: Academic Press. p. 409.

LIN, J. S. L., HUBER, N. & MURPHY, W. H. (1969) Immunization of C58 Mice to Line $I_b$ Leukemia. *Cancer Res.*, **29**, 2157.

PARISH, C. R. (1971*a*) Immune Response to Chemically Modified Flagellin. I. Induction of Antibody Tolerance to Flagellin by Acetoacetylated Derivatives of the Protein. *J. exp. Med.*, **134**, 1.

PARISH, C. R. (1971*b*) Immune Response to Chemically Modified Flagellin. II. Evidence for a Fundamental Relationship between Humoral and Cell Mediated Immunity. *J. exp. Med.*, **134**, 21.

RÉVÉSZ, L. (1960) Detection of Antigenic Differences in Isologous Host–Tumor Systems by Pretreatment with Heavily Irradiated Tumor Cells. *Cancer Res.*, **20**, 443.

ROUSE, B. T., ROLLINGHOFF, M. & WARNER, N. L. (1973) Tumour Immunity to Murine Plasma Cell Tumours. *Eur. J. Immun.*, **3**, 218.

SHIRRMACHER, V. & WIGZELL, H. (1972) Immune Responses Against Native and Chemically Modified Albumins in Mice. *J. exp. Med.*, **136**, 1616.

SIMMONS, R. L. & RIOS, A. (1971) Immunotherapy of Cancer: Immunospecific Rejection of Tumor in Recipients of Neuraminidase treated Tumor Cells plus BCG. *Science, NY.*, **174**, 591.

THOMPSON, K., HARRIS, M., BENJAMINI, E., MITCHELL, G. & NOBLE, M. (1972) Cellular and Humoral Immunity: A Distinction in Antigenic Recognition. *Nature, New Biol.*, **238**, 20.

WHITNEY, R. B., LEVY, J. G. & SMITH, A. G. (1974) The Influence of Tumor Size and Surgical Resection on Cell-mediated Immunity in Mice. *J. natn. Cancer Inst.* In the press.

ZBAR, B. & TANAKA, T. (1971) Immunotherapy of Cancer: Regression of Tumors after Intralesional Injection of Living *Mycobacterium bovis*. *Science, N.Y.* **172**, 271.

ZBAR, B., BERNSTEIN, I. D., BARTLETT, G. L., HANNA, M. G. & RAPP, H. J. (1972) Immunotherapy of Cancer: Regression of Intradermal Tumors and Prevention of Growth of Lymph Node Metastases after Intralesional Injection of Living *Mycobacterium bovis*. *J. natn. Cancer Inst.*, **91**, 119.